# Genomic *NGFB *variation and multiple sclerosis in a case control study

**DOI:** 10.1186/1471-2350-9-107

**Published:** 2008-12-08

**Authors:** Denis A Akkad, Niels Kruse, Larissa Arning, Ralf Gold, Jörg T Epplen

**Affiliations:** 1 Department of Human Genetics, Ruhr-University, 44780 Bochum, Germany; 2 IGSN, International Graduated School of Neuroscience, Ruhr-University, 44780 Bochum, Germany; 3 Institute for Multiple Sclerosis Research, University of Goettingen and Gemeinnuetzige Hertie-Stiftung, 37073 Goettingen, Germany; 4 Department of Neurology, St. Josef-Hospital, Ruhr-University, 44791 Bochum, Germany

## Abstract

**Background:**

Nerve growth factor β (NGFB) is involved in cell proliferation and survival, and it is a mediator of the immune response. ProNGF, the precursor protein of NGFB, has been shown to induce cell death via interaction with the p75 neurotrophin receptor. In addition, this neurotrophin is differentially expressed in males and females. Hence NGFB is a good candidate to influence the course of multiple sclerosis (MS), much like in the murine model of experimental autoimmune encephalomyelitis (EAE).

**Methods:**

Ten single nucleotide polymorphisms (SNPs) were genotyped in the *NGFB *gene in up to 1120 unrelated MS patients and 869 controls. Expression analyses were performed for selected MS patients in order to elucidate the possible functional relevance of the SNPs.

**Results:**

Significant association of NGFB variations with MS is evident for two SNPs. *NGFB *mRNA seems to be expressed in sex- and disease progression-related manner in peripheral blood mononuclear cells.

**Conclusion:**

NGFB variation and expression levels appear as modulating factors in the development of MS.

## Background

Multiple sclerosis (MS) was shown to depend on genetic components in various twin-, family- and association based studies[1]. This common neuro-inflammatory disease with neurodegenerative aspects leads to demyelination in the central nervous system (CNS)[1]. Complex interplays of environment and genetic factors are likely causes for disease development[2]. Yet, it is not entirely clear why females are usually affected more frequently than men. In different populations the only virtually consistent MS susceptibility factor is comprised in the *HLA-DRB1 *region. Recent studies point to complex allelic interactions of the *HLA-DRB1 *locus[3] underscoring possible (auto-) immune mechanisms in the pathogenesis of MS. Genetic factors determining susceptibility/disease progression are only incompletely understood, among which neurotrophins may be relevant like *e.g*. ciliary neurotrophic factors.

Here, we focussed on the nerve growth factor beta (*NGFB*) gene known to be involved in many cell regulatory pathways including cell survival and proliferation as well as in immune regulatory processes[4]. Analyses of NGFB in rodents[5] as well as in humans[6] show sex specific differences in secretion levels of the protein, whereby females have generally lower NGFB protein levels than males. In addition to crucial involvement in neuro-regulatory aspects of NGFB, the gene is located on chromosome 1p13.1, a region reported to be associated with MS, as revealed by admixture mapping[7]. NGFB has also been shown to delay the onset of clinical experimental autoimmune encephalomyelitis (EAE) as well as to prevent full development of EAE lesions[8]. Furthermore, anti-NGF mice reveal a progressive neurodegenerative phenotype[9]. Thus, altogether, *NGFB *is a prominent candidate gene to influence MS development.

## Methods

### Samples

DNA samples were genotyped from 1120 unrelated MS patients (372 males with 31.7 ± 9.8 years at age of onset and 748 females with 31.5 ± 10.0 years at age of onset), 622 of which showed relapsing remitting (rr), 252 secondary progressive (sp) and 249 primary progressive (pp) course according to the Poser criteria as well as 869 healthy blood donors (444 males, 47.9 ± 13.8 years of age and 425 females, 48.5 ± 16.1 years of age) resided in the Rhein-Ruhr area (Germany) as detailed in previous studies[10]. Informed consent has been obtained from all patients and controls. Research on human DNA for MS was approved by the ethics commission of the medical faculty of the Ruhr-University Bochum, Germany.

### Genotyping

Genotyping was performed via polymerase chain reaction (PCR) with subsequent restriction fragment length polymorphism (RFLP) analyses or via TaqMan^® ^assays (see additional file 1, table 3 for detailed information). Statistical analyses were done by χ^2 ^testing with a Bonferroni corrected statistical significance level. Hardy-Weinberg equilibrium was evaluated using Pearson's goodness-of-fit chi-square test (degree of freedom = 1). Power analyses for this study were performed using GPower software assuming small effect size of 0.20 of the variation, α = 0.05 and degree of freedom (DF) = 2 (data not shown)[11].

### Expression analyses

Fresh peripheral blood mononuclear cells (PBMCs) were obtained from MS patients in stable phases of the disease. 66% of patients received immunomodulatory treatment (Copaxone, MX, Betaferon and Cortison). RNA of 23 male patients (of which eight suffered from rr (three Ø treatment), eleven from sp (three Ø treatment) and four from pp MS (one Ø treatment)) as well as ten females (of which five showed rr (two Ø treatment) and five sp (two Ø treatment) MS course) was isolated using the RNeasy kit (Qiagen). Expression analyses were performed using QuantiTect^® ^SYBR^® ^Green one-step RT-PCR (Qiagen). The quantification of *NGFB *RNA was analysed using the ΔΔCt method. Oligonucleotides used were F-GCT TTC TAT CCT GGC CAC A, R-CAG GGA CAT TGC TCT CTG AG for the analysed NGFB as well as F-AGG TCG GAG TCA ACG GAT TTG, R-AAG CAG CCC TGG TGA CCA G for GAPDH as reference system. Statistical analyses were performed using t-tests (statistical program for the social sciences; SPSS).

## Results

**Figure 1:**
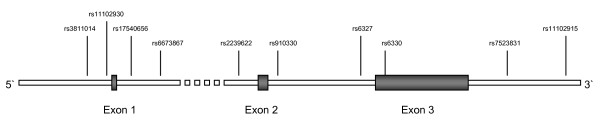
Schematic representation of the *NGFB *gene structure and the location of the genotyped SNPs.

We report genotyping data of ten SNPs covering the coding region of the *NGFB *gene with subsequent expression analysis in 23 male and ten female MS patients. A map of the genotyped SNPs in the *NGFB *gene is depicted in figure 1. Initial genotyping was performed for rs6330 located in exon 3 of the *NGFB *gene, since this exon encodes the precursor protein proNGF exclusively. rs6330 showed significant association in allele (p = 0.0038; OR = 1.210 (1.063–1.377)) and genotype frequencies (p = 0.0126) when comparing healthy controls and MS patients. Interestingly, the strongest association was observed mainly for male rr MS patients for allele (p = 0.0087; OR = 1.386 (1.086–1.770)) and genotype frequencies (p = 0.0230) when stratifying the cohorts by sex and disease progression, although the C allele was in general increased in all MS sub-cohorts (see table 1). Subsequent genotyping of three flanking SNPs (rs6327, rs7523831 and rs11102915) in subgroups of our cohorts of 263 rr MS patients and 259 controls showed no additional significant association (see additional file 1, table 4), thus restricting the region of interest. Further SNP genotyping in the vicinity of exons 1 and 2 was performed in order to clarify putative importance of these non-protein coding regions. No significant association was found for SNPs rs2239622 and rs910330 flanking exon 2 in the subgroups of our cohorts (see additional file 1, table 4).

**Table 1:** Detailed analysis for rs6330, genotyped in 1120 and 869 controls.

							Genotype (%)				p value				
SNP (maj./min. allele)	cohort	Stratif-ication	Maj. allele (%)	Min. allele (%)	p value	OR (CI*^1^)	XX	Xx	xx	Geno-type	Major allel dominant	co-dominant	Minor allele dominant	HWE p value	N
rs6330 (C/T)	MS	Ø	1235 (57.2)	923 (42.8)	**0.0038**	**1.210** **(1.063–1.377)**	356 (33.0)	523 (48.5)	200 (18.5)	**0.0126**	0.0579	0.3034	**0.0056**	0.75	1079
		male	438 (59.8)	294 (40.2)	**0.0087**	**1.308** **(1.070–1.599)**	133 (36.3)	172 (47.0)	61 (16.7)	**0.0247**	0.1109	0.2403	0.0091	0.67	366
		female	797 (55.9)	629 (44.1)	0.0598	1.149 (0.946–1.395)	223 (31.3)	351 (49.2)	139 (19.5)	0.1672	0.1665	0.6840	0.0938	0.97	713
		rr Ø	677 (56.9)	513 (43.1)	**0.0211**	**1.193** **(1.015–1.210)**	192 (32.3)	293 (49.2)	110 (18.5)	0.0647	0.1011	0.5493	0.0345	0.92	595
		rr male	240 (61.2)	152 (38.8)	**0.0087**	**1.386** **(1.086–1.770)**	75 (38.3)	90 (45.9)	31 (15.8)	**0.0230**	0.1196	0.2226	**0.0081**	0.65	196
		rr female	437 (54.8)	361 (45.2)	0.2276	1.098 (0.885–1.361)	117 (29.3)	203 (50.9)	79 (19.8)	0.4648	0.2701	0.9151	0.3737	0.59	399
		sp Ø	271 (56.5)	209 (43.5)	0.1276	1.173 (0.955–1.439)	78 (32.5)	115 (47.9)	47 (19.6)	0.2503	0.4119	0.4231	0.1016	0.69	240
		sp male	73 (55.3)	59 (44.7)	0.6597	1.086 (0.752–1.569)	21 (31.8)	31 (47.0)	14 (21.2)	0.7583	0.9914	0.5229	0.4839	0.69	66
		sp female	198 (56.9)	150 (43.1)	0.1082	1.197 (0.915–1.565)	57 (32.8)	84 (48.3)	33 (19.0)	0.2593	0.2818	0.6243	0.1264	0.84	174
		pp Ø	287 (58.8)	201 (41.2)	**0.0143**	**1.291** **(1.052–1.584)**	86 (35.2)	115 (47.1)	43 (17.6)	0.0369	0.1358	0.3070	**0.0137**	0.67	244
		pp male	125 (60.1)	83 (39.9)	0.0759	1.322 (0.971–1.800)	37 (35.6)	51 (49.0)	16 (15.4)	0.1967	0.1883	0.6930	0.1116	0.82	104
		pp female	162 (57.9)	118 (42.1)	0.0779	1.245 (0.933–1.660)	49 (35.0)	64 (45.7)	27 (19.3)	0.1540	0.3615	0.3297	0.0557	0.46	140
	control	Ø	857 (52.5)	775 (47.5)			221 (27.1)	415 (50.9)	180 (22.1)					0.57	816
		male	443 (53.2)	389 (46.8)			115 (27.6)	213 (51.2)	88 (21.2)					0.56	416
		female	414 (51.7)	386 (48.3)			106 (26.5)	202 (50.5)	92 (23.0)					0.82	400

Significance threshold: p = 0.025 (Bonferroni corrected for 2 SNPs). Significant values indicated by bold letter;

^1^CI = 95%. HWE: Hardy-Weinberg equilibrium, Pearson's goodness-of-fit chi-square (degree of freedom = 1).

A tendency for MS association of the promoter SNP rs11102930 was observed for the co-dominant transmission model for the subgroup of 263 rr MS patients as compared to healthy controls (see additional file 1, table 4). Gender stratification showed, despite of decreased power, significant association of rs11102930 in male MS patients compared to male controls for the co-dominant transmission model (data not shown). Female MS patients did not show conspicuous differences when compared to healthy female controls. Analyses of three flanking SNPs, rs3811014, rs17540656 and rs6673867 in the defined subgroups showed no significant association for male MS patients compared to healthy male controls as well as no significant association between female MS patients and female controls, respectively (data not shown). The conspicuous tendency of sex-specific association of rs11102930 SNP in male MS patients was confirmed when additionally the remaining individuals were genotyped (860 unrelated MS patients and 611 controls, p = 0.0115; OR = 0.765 (0.622–0.942)). Further stratification of all tested MS patients for disease progression indicates despite the small male rr MS cohort size (n = 179) that the significant association is eventually based on the difference in allele (p = 0.0101; OR = 0.717 (0.557–0.924)) and genotype (p = 0.0206) frequency distributions of male rr MS patients compared to healthy male controls (see table 2).

**Table 2:** Detailed analysis for rs11102930, genotyped in 1120 and 869 controls.

							Genotype (%)				p value				
SNP (maj./min. allele)	cohort	Stratif-ication	Maj. allele (%)	Min. allele (%)	p value	OR (CI*^1^)	XX	Xx	xx	Geno-type	Major allel dominant	co-dominant	Minor allele dominant	HWE p value	N
rs11102930 (T/C)	MS	Ø	1380 (64.3)	766 (35.7)	0.5414	0.959 (0.840–1.096)	449 (41.8)	482 (44.9)	142 (13.2)	0.6694	0.3713	0.7157	0.8148	0.48	1073
		male	423 (60.8)	273 (39.2)	**0.0115**	**0.765 (0.622–0.942)**	124 (35.6)	175 (50.3)	49 (14.1)	0.0259	0.2272	0.0664	**0.0075**	0.31	348
		female	957 (66.0)	493 (34.0)	0.2271	1.115 (0.934–1.332)	325 (44.8)	307 (42.3)	93 (12.8)	0.1682	0.8954	0.0695	0.0829	0.13	725
		rr Ø	764 (65.1)	410 (34.9)	0.9120	0.991 (0.848–1.158)	251 (42.8)	262 (44.6)	74 (12.6)	0.8773	0.6845	0.7010	0.9061	0.66	587
		rr male	212 (59.2)	146 (40.8)	**0.0101**	**0.717 (0.557–0.924)**	59 (33.0)	94 (52.5)	26 (14.5)	**0.0206**	0.2537	0.0465	**0.0056**	0.24	179
		rr female	552 (67.6)	264 (32.4)	0.0760	1.201 (0.981–1.472)	192 (47.1)	168 (41.2)	48 (11.8)	0.0872	0.7263	0.0525	0.0295	0.23	408
		sp Ø	303 (62.9)	179 (37.1)	0.3324	0.901 (0.731–1.112)	97 (40.2)	109 (45.2)	35 (14.5)	0.5288	0.2712	0.8855	0.5545	0.63	241
		sp male	78 (59.1)	54 (40.9)	0.0764	0.714 (0.491–1.036)	23 (34.8)	32 (48.5)	11 (16.7)	0.2125	0.2026	0.4665	0.1183	0.98	66
		sp female	225 (64.3)	125 (35.7)	0.7989	1.034 (0.798–1.341)	74 (42.3)	77 (44.0)	24 (13.7)	0.6875	0.7015	0.3886	0.5387	0.58	175
		pp Ø	313 (63.9)	177 (36.1)	0.5743	0.942 (0.764–1.161)	101 (41.2)	111 (45.3)	33 (13.5)	0.7926	0.5014	0.9019	0.7477	0.77	245
		pp male	133 (64.6)	73 (35.4)	0.5167	0.900 (0.655–1.237)	42 (40.8)	49 (47.6)	12 (11.7)	0.7250	0.8995	0.4775	0.4289	0.69	103
		pp female	180 (63.4)	104 (36.6)	0.9694	0.995 (0.753–1.314)	59 (41.5)	62 (43.7)	21 (14.8)	0.6352	0.4961	0.3850	0.6777	0.48	142
	control	Ø	1121 (65.3)	597 (34.7)			364 (42.4)	393 (45.8)	102 (11.9)					0.79	859
		male	585 (66.9)	289 (33.1)			197 (45.1)	191 (43.7)	49 (11.2)					0.79	437
		female	536 (63.5)	308 (36.5)			167 (39.6)	202 (47.9)	53 (12.6)					0.50	422

Significance threshold: p = 0.025 (Bonferroni corrected for 2 SNPs). Significant values indicated by bold letter;

^1^CI = 95%. HWE: Hardy-Weinberg equilibrium, Pearson's goodness-of-fit chi-square (degree of freedom = 1).

**Figure 2:**
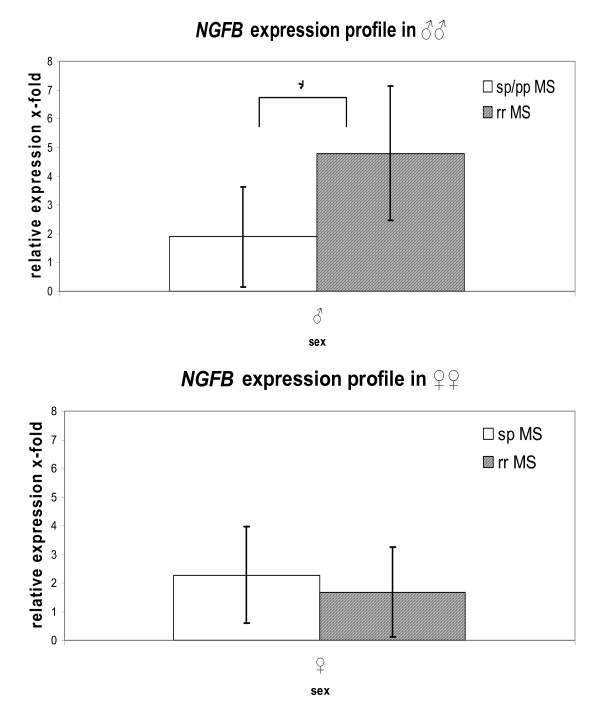
**NGFB expression profile comparison of male rr *vs*. sp/pp MS patients and female rr *vs*. sp MS patients, respectively.** Significant differences are obvious between male MS patients with different course of the disease; *p = 0.003 (SPS t-test).

23 male MS patients were characterized via quantitative RT-PCR after selection for opposite homozygosity in rs11102930: No correlation was observed between genotypes and expression levels. Yet, correlating with disease progression reveals significant differences in NGFB RNA expression levels between rr and sp/pp MS patients, respectively (p = 0.003; see figure 2). We evaluated the NGFB expression levels in correlation with disease course also in females, although no differences were observed in the allele and genotype frequencies for rs11102930. Five female patients with rr MS were compared to five female patients with sp course, both harboring the more frequent homozygous genotype for rs11102930. The two groups showed no tendencies for different expression profiles. Influence of MS treatment on the observed expression data was not evident.

## Discussion

NGFB as well as its precursor protein proNGF have been shown to be involved in several crucial processes relating to MS pathogenesis. NGFB itself, in addition to its important role as modulatory mediator of the immune response[12], initiates and maintains cell proliferation and survival. In contrast, proNGF has been shown to induce cell death via interaction with the p75 neurotrophin receptor[13]. Here we present genotyping data of the *NGFB *gene for ten selected SNPs in up to 1120 unrelated MS patients and 869 controls. Of ten tested SNPs two show significant association in sex- and disease progression-specific manner, whereby rs6330 is located in exon 3 and rs11102930 in the promoter region of the *NGFB *gene (see figure 1). SNP rs6330 is defined as a C/T exchange leading to the non-synonymous amino acid substitution of alanine to valine in position 35. The increase in amino acid size could modify the tertiary structure of proNGF, leading to altered interaction and signalling via the p75 neurotrophin receptor. Since the C allele is more frequent in MS patients compared to healthy controls (57.2% *vs*. 52.5%), this allele could represent a risk factor due to its possible role in inducing increased cell death via enhanced interaction with the p75 neurotrophin receptor. Interestingly, male MS patients seem to have a higher risk than female MS patients (see table 1). The actual effect of the amino acid exchange on eventually altered interaction of proNGF and the p75 neurotrophin receptor has to be shown definitively in follow-up studies in order to clarify any functional relevance.

rs11102930 is located in the promoter region of the *NGFB *gene and has been shown via electrophoretic mobility shift assays to be functionally relevant by affecting the binding of the vitamin D receptor (VDR) to its DNA motif[14]. VDR has a higher binding affinity for the C than for the T allele. Interestingly, VDR variation itself has been found to be associated with MS [15] and it affects the EAE course in the mouse model[16]. For our MS cohort, we show that only male patients have a significantly increased frequency of the C allele p_unc _= 0.01 and a significant effect in the C dominant model for the genotype distribution p_unc _= 0.0075 (see table 2). Further stratification of male MS patients for rs11102930 association indicates that only male rr MS patients show a significantly increased frequency of the C allele p_unc _= 0.01. This effect has to be verified in larger male (rr) MS cohorts in order to provide convincing statistical power. Under the assumption of enhanced NGFB expression due to the higher affinity of the VDR binding in case of the C allele, we propose a protective effect of the C allele due to higher NGFB levels and its cell proliferating and survival mediating properties, thus preventing chronic disease progression. Although rs11102930 is reported to alter the binding affinity of the VDR, the expression regulatory influence has still to be shown via *in vitro *reporter assays. Our analyses of the rs11102930 genotype on the expression profile revealed no correlation of the genotype patterns for SNP rs11102930 and NGFB levels. Yet, male rr MS patients have a 2.5 fold elevated expression in comparison to male sp + pp MS patients as shown in figure 2 (or only male sp MS, data not shown). Both, sp and pp MS patients were evaluated in combination because of chronic disease progression. This expression difference based on disease progression differentiation was not observed in female MS patients perhaps due to the small sample size, differences in menstruation cycle stages (no information available) or perhaps there are no differences. Comparison between female and male MS patients were not feasible since sex-specific expression differences have been described for healthy controls[5,6].

Our results indicate a disease modulating role of NGFB for MS progression as shown in the EAE model[8]. The previously reported lower NGFB levels in healthy females compared to healthy males could perhaps partially explain why females are affected twice as often as males with MS based on the neuro-protective and cell proliferative stimulating properties of NGFB. Since the serum level of NGFB is low in females and, in addition, depends on the menstrual cycle phase[17], marginal modulations of NGFB expression or NGFB interactions may have no profound effects on disease progression in women. This fact could explain why no significant differences in genotype and allele frequency patterns for rs6330 and rs11102930 were observed in the analysed female MS patients. Under this assumption we hypothesise in the case of rs6330, the C allele to be a risk factor for MS by eventually modulating the interaction of the proNGF with the p75 neurotrophin receptor and thus promoting cell death. In contrast the rs11102930 C allele located in the promoter region might provide protection for male MS patients, eventually preventing chronic disease progression due to perhaps increased NGFB expression. Which of the significantly associated SNPs are responsible for the change in expression profile of NGFB? No correlation could be drawn between the expression results and the genotype pattern. Hence, it is likely that combined effects of the tested SNPs lead to the observed results. According to our expression lower NGFB concentration in male MS patients may lead to chronic disease progression (see figure 2). Such an assertion needs to be verified independently.

The complex LD patterns for the 52.32 kilobase pair long sequence (see HapMap data) and the haplotype analysis for the 10 tested SNPs make predictions of putative interactions of rs6330 with rs11102930 largely impossible (see additional file 1, figure 3 for detailed information). Conversely, it is theoretically possible that other linked sequence variations are responsible for the observed phenomena.

## Conclusion

NGFB as multifunctional protein might affect MS courses differentially as well as other neurodegenerative diseases in a complex manner. Therefore, further detailed studies appear warranted.

## Competing interests

The authors declare that they have no competing interests.

## Authors' contributions

DAA performed the genotyping, calculated the statistics and drafted the manuscript. NK performed the expression analyses. LA, RG and JTE participated in the design of the study, evaluated the data and finalised the manuscript which was approved by all authors in the final version.

## Pre-publication history

The pre-publication history for this paper can be accessed here:



## Supplementary Material

Additional File 1 **Assay data and statistical analysis (Chi-Square, Haplotype block representation) for the 10 tested *NGFB *SNPs.** Provided are the data for the genotyping assays of the 10 tested SNPs in the *NGFB *gene as well as the corresponding statistical analysis including the Haplotype block representation. Click here for file
