# Supplementary material for: Genomic NGFB variation and multiple sclerosis in a case control study
Source: BMC Med Genet. 2008 Dec 8;9:107. doi: 10.1186/1471-2350-9-107 (PMC2613874; doi:10.1186/1471-2350-9-107)
Supplement: Additional File 1 — Assay data and statistical analysis (Chi-Square, Haplotype block representation) for the 10 tested NGFB SNPs. Provided are the data for the genotyping assays of the 10 tested SNPs in the NGFB gene as well as the corresponding statistical analysis including the Haplotype block representation. [file 1471-2350-9-107-S1.doc]

Additional file

Additional file, table 3: Oligonucleotide, restriction enzyme and TaqMan® assay information for the 10 tested SNPs in the *NGFB* gene.

| SNP | System | Primer | Restriction Enzyme |
| --- | --- | --- | --- |
| rs3811014 | RFLP | F- TGAATGATGCCACAAGAACAC  R- AGGCTTGTCCACTTCTGACTT | TspRI |
| rs11102930 | TaqMan | C__26680904_10 | - |
| rs17540656 | RFLP | F- GTAAAACGACGGCCAGTGTAGAGGTCGCCAAGGTACAG  R- CAGGAAACAGCTATGACAGGGAGCTAAGCTGCTTGTT | TaaI |
| rs6673867 | RFLP | F- GTAAAACGACGGCCAGTAGATGTCAGTGCCAGACCTG  R- CAGGAAACAGCTATGACTGGGTTGTTTGCACACAGA | MboI |
| rs2239622 | RFLP | F- CGATGGTGGCTTTTCTGAC  R- GTGGAGGGCTTTGAACAAG | NlaIII |
| rs910330 | RFLP | F- TTGCATATTTCTATCATGGGC  R- AAGCCGTATGCACACTGTAAC | HaeIII |
| rs6327 | RFLP | F- CAGGAAACAGCTATGACTTGTGCCTTGATGTGATGG  R- CAGGAAACAGCTATGACTTGTGCCTTGATGTGATGG | SchI |
| rs6330 | RFLP | F- GTAAAACGACGGCCAGTCTCTGAGGGACCCAGAAACT  R- CAGGAAACAGCTATGACAGTGTCAAGGGAATGCTGAA | Hpy8I |
| rs7523831 | TaqMan | C__25928712_10 | - |
| rs11102915 | RFLP | F- AAGTTGGCTCCTTTCTCAGC  R- GGGTGATCTGAATATGCGATT | HpaII |

|  |  |  |  |  |  |  | Genotype (%) |  |  | p value |  |  |  |
| --- | --- | --- | --- | --- | --- | --- | --- | --- | --- | --- | --- | --- | --- |
| SNP  (Maj. / Min. allele) | cohort | stratification | Maj. allele (%) | Min. allele (%) | p value | XX | Xx | xx | Maj. allele dominant | co-dominant | Min. allele dominant | HWE p value | N |
| rs3811014 (A/G) | MS | Ø | 400 (78.4) | 110 (21.6) | 0.6659 | 158 (62.0) | 84 (32.9) | 13 (5.1) | 0.9603 | 0.5616 | 0.5882 | 0.68 | 255 |
|  | control | Ø | 371 (77.3) | 109 (22.7) |  | 143 (59.6) | 85 (35.4) | 12 (5.0) |  |  |  | 0.89 | 240 |
| rs11102930 (T/C) | MS | Ø | 329 (63.0) | 193 (37.0) | 0.3056 | 101 (38.7) | 127 (48.7) | 33 (12.6) | 0.7164 | 0.0589 | 0.0985 | 0.48 | 261 |
|  | control | Ø | 337 (66.1) | 173 (33.9) |  | 117 (45.9) | 103 (40.4) | 35 (13.7) |  |  |  | 0.12 | 255 |
| rs17540656 (A/G) | MS | Ø | 281 (54.2) | 237 (45.8) | 0.2069 | 76 (29.3) | 129 (49.8) | 54 (20.8) | 0.4479 | 0.5704 | 0.2095 | 0.96 | 259 |
|  | control | Ø | 276 (58.2) | 198 (41.8) |  | 82 (34.6) | 112 (47.3) | 43 (18.1) |  |  |  | 0.66 | 237 |
| rs6673867 (G/T) | MS | Ø | 262 (50.4) | 258 (49.6) | 0.6305 | 66 (25.4) | 130 (50.0) | 64 (24.6) | 0.5897 | 0.8131 | 0.8000 | 0.99 | 260 |
|  | control | Ø | 244 (51.9) | 226 (48.1) |  | 62 (26.4) | 120 (51.1) | 53 (22.6) |  |  |  | 0.73 | 235 |
| rs2239622 (C/T) | MS | Ø | 392 (76.6) | 120 (23.4) | 0.6586 | 152 (59.4) | 88 (34.4) | 16 (6.3) | 0.6350 | 0.9309 | 0.7567 | 0.50 | 256 |
|  | control | Ø | 384 (77.7) | 110 (22.3) |  | 150 (60.7) | 84 (34.0) | 13 (5.3) |  |  |  | 0.78 | 247 |
| rs910330 (C/A) | MS | Ø | 384 (73.8) | 136 (26.2) | 0.4467 | 140 (53.8) | 104 (40.0) | 16 (6.2) | 0.7160 | 0.5421 | 0.4422 | 0.56 | 260 |
|  | control | Ø | 366 (75.9) | 116 (24.1) |  | 138 (57.3) | 90 (37.3) | 13 (5.4) |  |  |  | 0.73 | 241 |
| rs6327 (G/A) | MS | Ø | 301 (57.4) | 223 (42.6) | 0.1127 | 83 (31.7) | 135 (51.5) | 44 (16.8) | 0.1746 | 0.9226 | 0.2001 | 0.38 | 262 |
|  | control | Ø | 269 (52.5) | 243 (47.5) |  | 68 (26.6) | 133 (52.0) | 55 (21.5) |  |  |  | 0.50 | 256 |
| rs7523831 (G/C) | MS | Ø | 337 (64.1) | 189 (35.9) | 0.8154 | 113 (43.0) | 111 (42.2) | 39 (14.8) | 0.2512 | 0.2144 | 0.6416 | 0.18 | 263 |
|  | control | Ø | 329 (64.8) | 179 (35.2) |  | 104 (40.9) | 121 (47.6) | 29 (11.4) |  |  |  | 0.48 | 254 |
| rs11102915 (T/C) | MS | Ø | 326 (64.4) | 180 (35.6) | 0.9501 | 110 (43.5) | 106 (41.9) | 37 (14.6) | 0.2969 | 0.1836 | 0.5309 | 0.17 | 253 |
|  | control | Ø | 305 (64.6) | 167 (35.4) |  | 96 (40.7) | 113 (47.9) | 27 (11.4) |  |  |  | 0.47 | 236 |

Additional file, table 4: *NGFB* genotyping for 9 SNPs in 263 rr MS patients and 259 controls. Significance threshold: p≤0.006 (Bonferroni corrected for 9 SNPs). HWE: Hardy-Weinberg equilibrium, Pearson's goodness-of-fit chi-square (degree of freedom = 1).


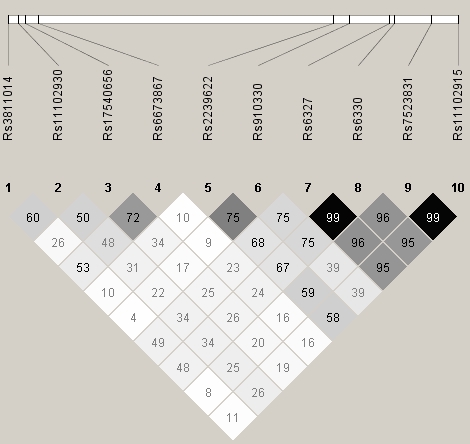


5’

3’

Exon 1

Exon 2

Exon 3

Additional file, figure 3: Haplotype block representation of the 10 investigated SNPs for the *NGFB* gene. Interactions between rs6330 with rs11102930 are not predictable as underscored by the HapMap project: <http://www.hapmap.org/cgi-perl/gbrowse/hapmap_B35/> Landmark: chr1:115,540,581-115,592,899.
